# Supplementary material for: An optimized framework for simultaneous EEG-fMRI at 7T enabling safe, high-quality human brain imaging with millisecond temporal resolution and sub-millimeter spatial resolution
Source: Imaging Neurosci (Camb). 2025 Nov 4;3:IMAG.a.983. doi: 10.1162/IMAG.a.983 (PMC12587055; doi:10.1162/IMAG.a.983)
Supplement: Supplementary Material [file IMAG.a.983_supp.pdf]

Supplementary Figures

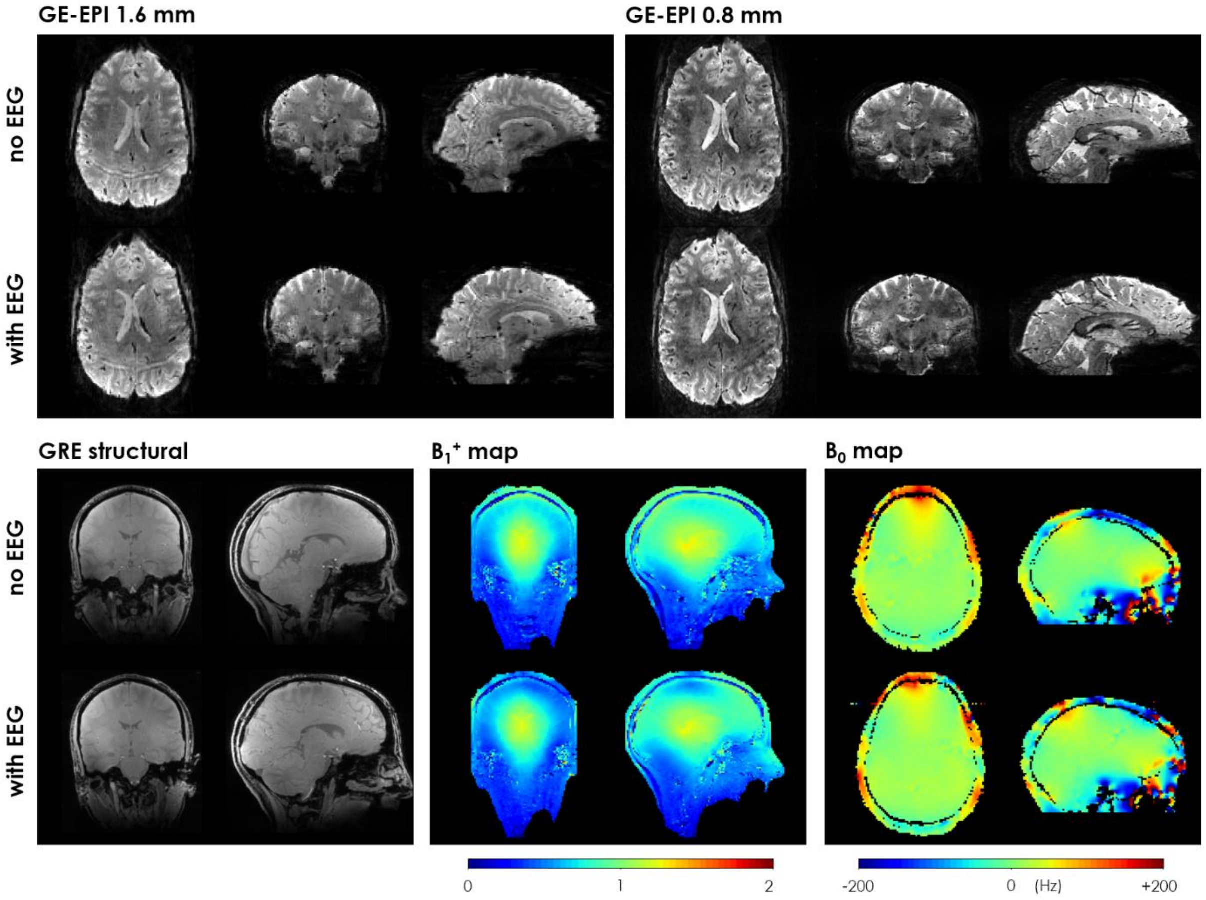

Supp. Fig. 1. MRI data obtained from an example subject with and without the EEG system in place. This example used the BrainCap MR7Flex prototype. The slices shown include some of the most relevant differences found between the with- and without-EEG conditions. Top: GE-EPI volumes from the fMRI acquisitions (left: 1.6mm isotropic resolution; right: 0.8 mm isotropic resolution). Bottom: GRE-based anatomical image and field maps. The B_1^+ map is expressed as a fraction of the nominal flip angle. Both maps were masked to exclude background voxels.

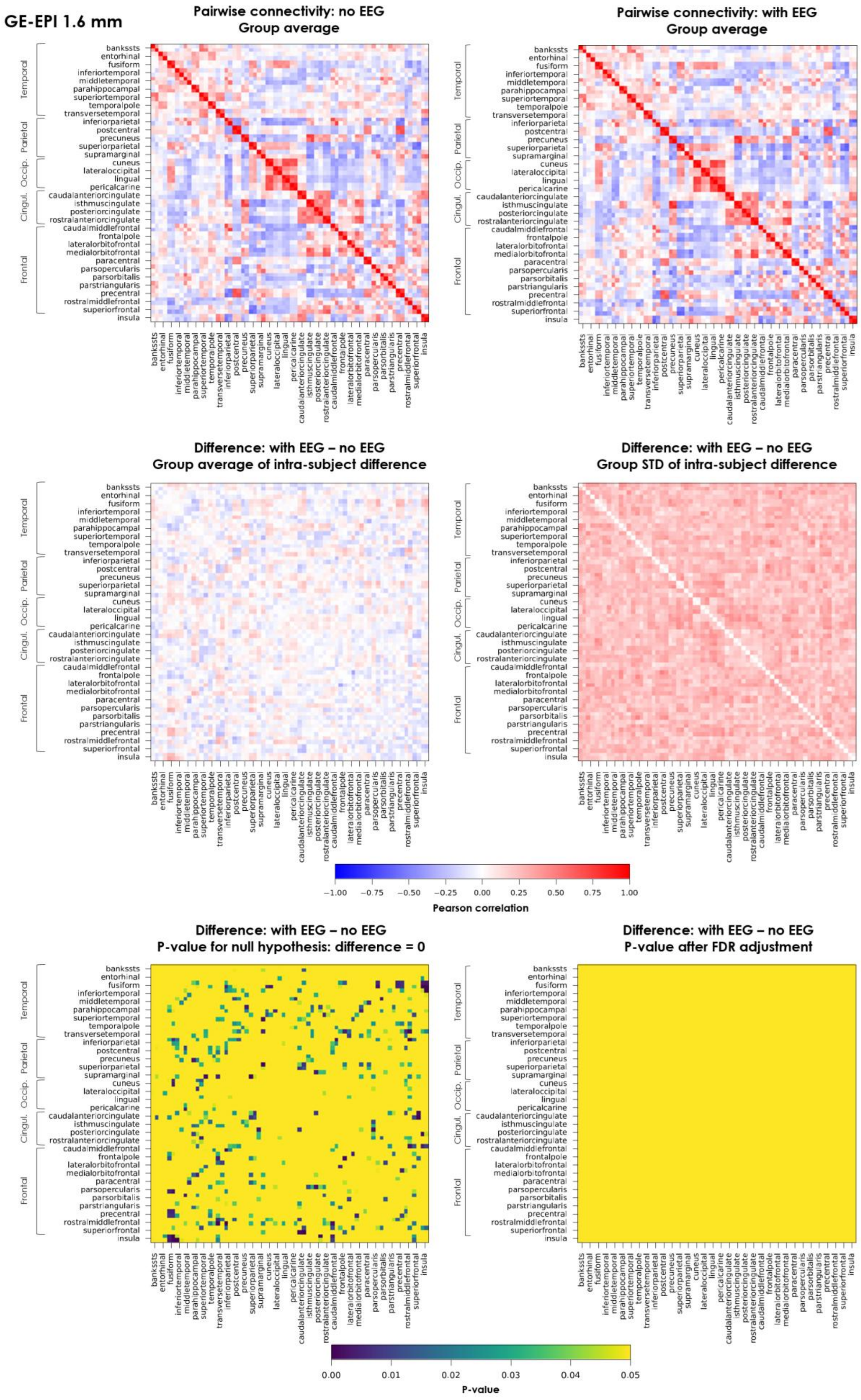
GE-EPI 1.6 mm
Pairwise connectivity: no EEG
Group average
Pairwise connectivity: with EEG
Group average
Difference: with EEG – no EEG
Group average of intra-subject difference
Difference: with EEG – no EEG
Group STD of intra-subject difference
Difference: with EEG – no EEG
P-value for null hypothesis: difference = 0
Difference: with EEG – no EEG
P-value after FDR adjustment
Temporal
Parietal
Occip.
Cingul.
Frontal
bankssts
entorhinal
fusiform
inferiortemporal
middletemporal
parahippocampal
superiortemporal
temporalpole
transversetemporal
inferiorparietal
postcentral
precuneus
superiorparietal
supramarginal
cuneus
lateraloccipital
lingual
pericalcarine
caudalanteriorcingulate
isthmuscingulate
posteriorcingulate
rostralanteriorcingulate
caudalmiddlefrontal
frontalpole
lateralorbitofrontal
medialorbitofrontal
paracentral
parsopercularis
parsorbitalis
parstriangularis
precentral
rostralmiddlefrontal
superiorfrontal
insula
−1.00
−0.75
−0.50
−0.25
0.00
0.25
0.50
0.75
1.00
Pearson correlation
0.00
0.01
0.02
0.03
0.04
0.05
P-value

Supp. Fig. 2. Impact of EEG on fMRI resting-state connectivity across different cortical region pairs (DK atlas), evaluated on the 1.6 mm GE-EPI acquisitions with the in-house EEG cap prototype. Top row: functional connectivity matrix, averaged across subjects, for the acquisitions without and with EEG. Prior to group averaging, the individual matrices were obtained following the approach described in (Wirsich et al., 2021), based on Peason correlation. Each region label is centered on a pair of rows (resp. columns) which refer to the left and the right hemisphere for the same region. Middle row: group average (left), and group standard deviation (right) of the difference matrices between without- and with-EEG acquisitions of each subject. Bottom row: p-values for the group-level statistical significance of a difference in functional connectivity between without and with EEG conditions, for each DK region pair; the p-values are shown without correction for multiple comparisons on the left, and after adjustment based on false discovery rate (FDR) (Benjamini and Hochberg, 1995) on the right (*false_discovery_control* function from SciPy, Python). The color scale is given an upper limit of p = 0.05, as a threshold for significance.

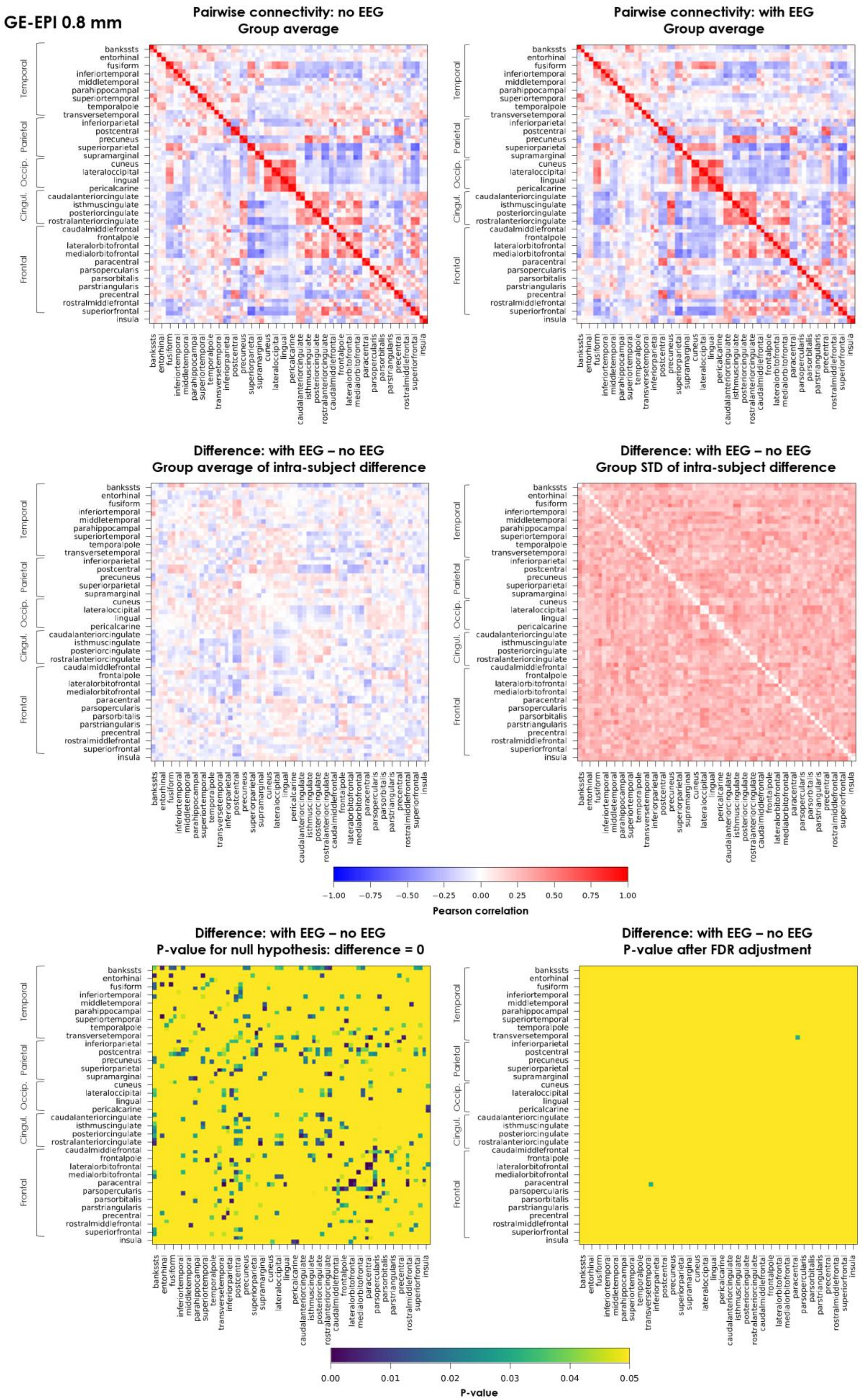
GE-EPI 0.8 mm
Pairwise connectivity: no EEG
Group average
Pairwise connectivity: with EEG
Group average
Difference: with EEG – no EEG
Group average of intra-subject difference
Difference: with EEG – no EEG
Group STD of intra-subject difference
Difference: with EEG – no EEG
P-value for null hypothesis: difference = 0
Difference: with EEG – no EEG
P-value after FDR adjustment
Temporal
Parietal
Occip.
Cingul.
Frontal
bankssts
entorhinal
fusiform
inferiortemporal
middletemporal
parahippocampal
superiortemporal
temporalpole
transversetemporal
inferiorparietal
postcentral
precuneus
superiorparietal
supramarginal
cuneus
lateraloccipital
lingual
pericalcarine
caudalanteriorcingulate
isthmuscingulate
posteriorcingulate
rostralanteriorcingulate
caudalmiddlefrontal
frontalpole
lateralorbitofrontal
medialorbitofrontal
paracentral
parsopercularis
parsorbitalis
parstriangularis
precentral
rostralmiddlefrontal
superiorfrontal
insula
−1.00 −0.75 −0.50 −0.25 0.00 0.25 0.50 0.75 1.00
Pearson correlation
0.00 0.01 0.02 0.03 0.04 0.05
P-value

Supp. Fig. 3. Impact of EEG on fMRI resting-state connectivity across different cortical region pairs (DK atlas), evaluated on the 0.8 mm GE-EPI acquisitions with the in-house EEG cap prototype. Top row: functional connectivity matrix, averaged across subjects, for the acquisitions without and with EEG. Prior to group averaging, the individual matrices were obtained following the approach described in (Wirsich et al., 2021), based on Peason correlation. Each region label is centered on a pair of rows (resp. columns) which refer to the left and the right hemisphere for the same region. Middle row: group average (left), and group standard deviation (right) of the difference matrices between without- and with-EEG acquisitions of each subject. Bottom row: p-values for the group-level statistical significance of a difference in functional connectivity between without and with EEG conditions, for each DK region pair; the p-values are shown without correction for multiple comparisons on the left, and after adjustment based on false discovery rate (FDR) (Benjamini and Hochberg, 1995) on the right (*false_discovery_control* function from SciPy, Python). The color scale is given an upper limit of p = 0.05, as a threshold for significance.

GE-EPI 1.6 mm

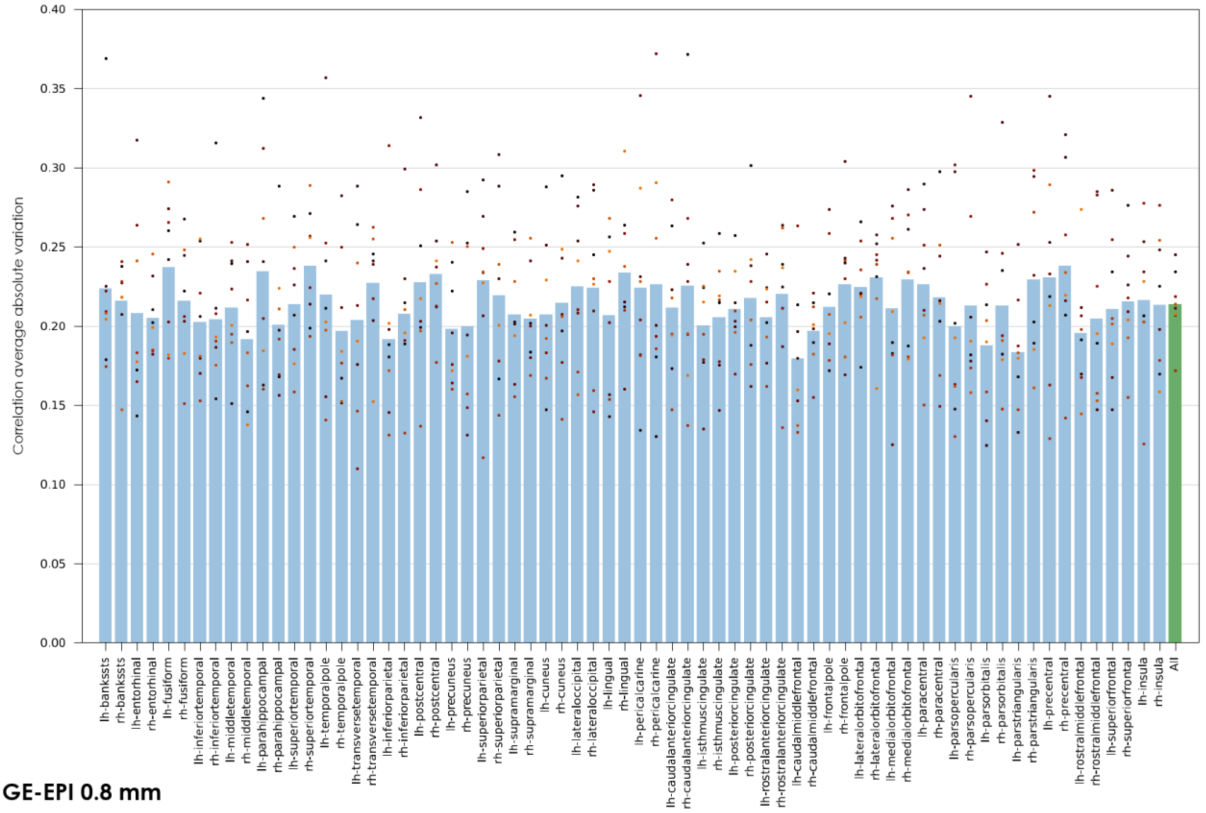

GE-EPI 0.8 mm

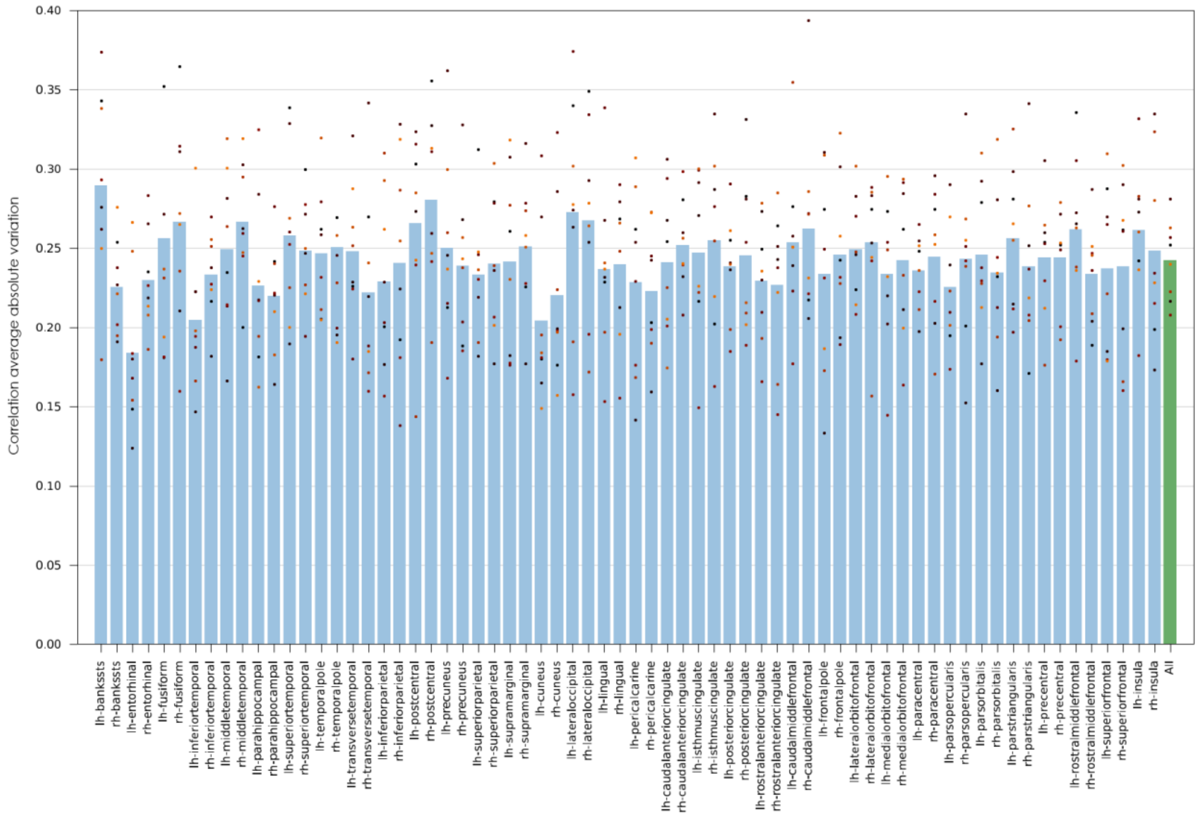

Supp. Fig. 4. Impact of EEG on fMRI resting-state connectivity across different cortical regions (DK atlas), evaluated on the 1.6 mm and 0.8 mm GE-EPI acquisitions with the in-house EEG cap prototype. Each dot marker for each DK region represents the absolute difference in Pearson correlation between without- and with-EEG conditions, averaged across all pairs that include that region, for one individual

subject; the bar represents the respective average across subjects. The Pearson correlation estimates were obtained following the approach described in (Wirsich et al., 2021). In the region labels, “lh” and “rh” indicate the left and right hemisphere, respectively.

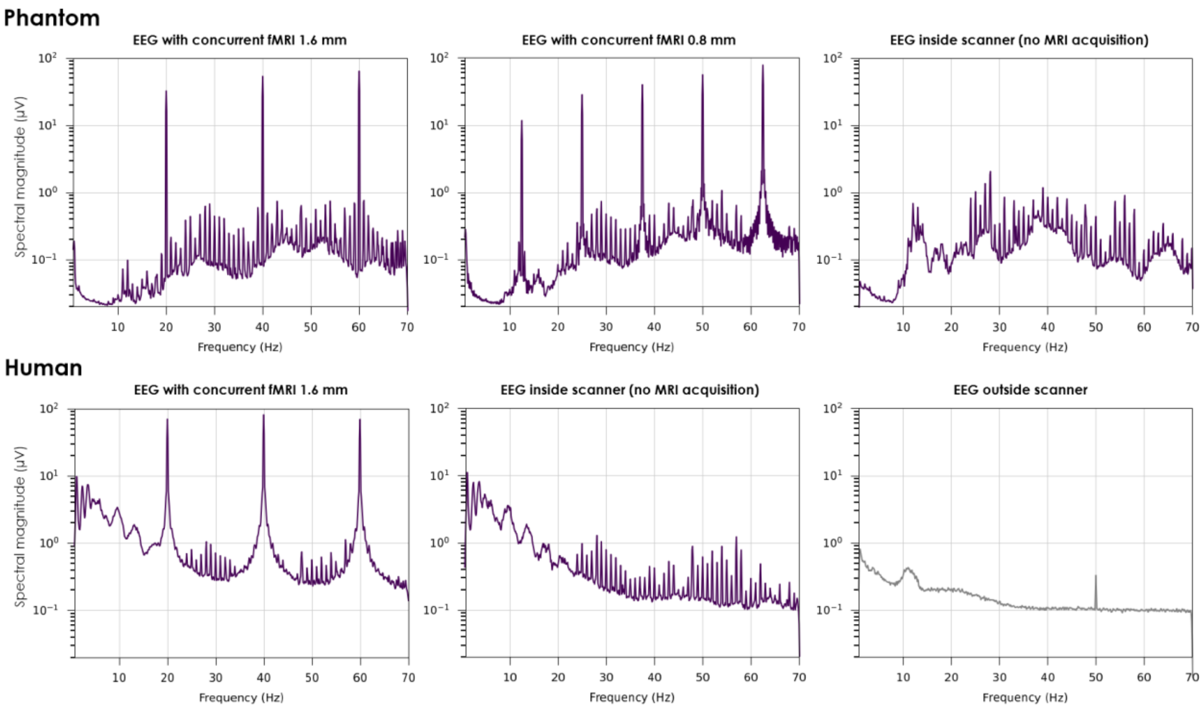

Supp. Fig. 5. Impact of different MRI-related artifacts on EEG recordings, in a phantom (top) and an example participant (bottom), with and without concurrent fMRI acquisitions, as well as outside the scanner in a non-shielded room (for the human participant). For consistency with the conditions shown in Fig. 6, the source EEG recordings were first downsampled to 200 Hz, bandpass-filtered to 0.75–70 Hz and re-referenced to the channel average. Magnitude spectra were then estimated using Welch’s method (10 s Hann window, 50% overlap) for each channel, and then averaged across channels. For reference, the slice GA peaks are expected at 20 Hz for the 1.6 mm fMRI protocol (respectively 12.5 Hz for the 0.8 mm protocol) and harmonics; the volume GA peak, if present, is expected at 0.95 Hz (respectively 0.28 Hz).

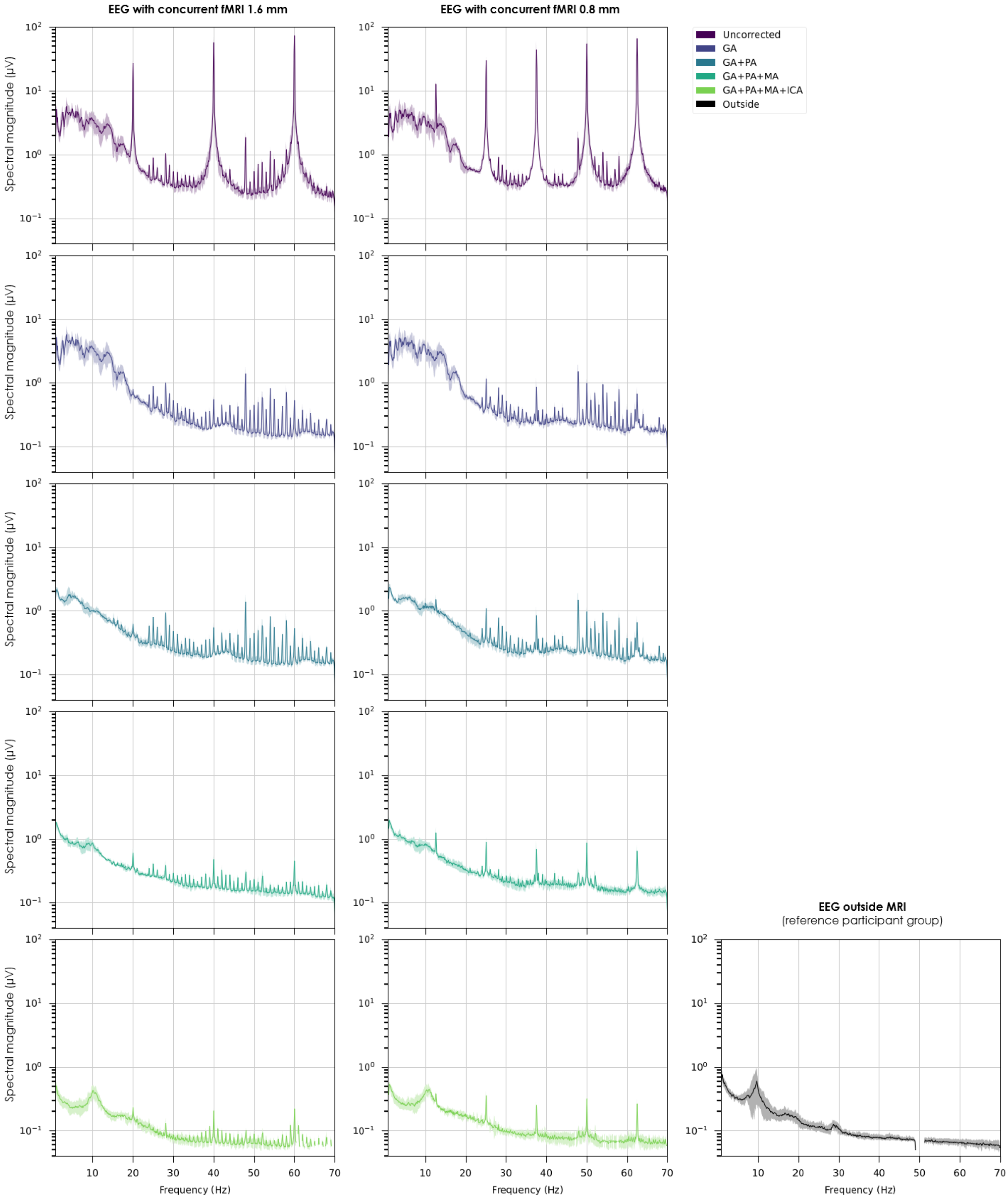

Supp. Fig. 6. Impact of MRI-related artifacts and the respective correction steps on EEG data quality, in terms of spectral content. Left and middle column: signal spectra for the EEG-fMRI group, with either fMRI protocol, after different correction steps; for consistency, all cases were first downsampled to 200 Hz, bandpass-filtered to 0.75–70 Hz and re-referenced to the channel average; magnitude spectra were then estimated using Welch's method (10 s Hann window, 50% overlap) for each channel, and averaged across channels. The colored lines represent the average of these signals across participants; the colored error margins represent the STD across participants. For reference, the slice GA peaks are expected at 20 Hz for the 1.6 mm fMRI protocol (respectively 12.5 Hz for the 0.8 mm protocol) and harmonics; the volume GA peak, if present, is expected at 0.95 Hz (respectively 0.28 Hz). Bottom-right: signal spectrum for the reference participant group recorded outside MRI (non-shielded room),

pre-processed in a similar manner, except for an additional 50 Hz notch filter; the black line denotes the average across participants; the gray error margin denotes the STD across participants.

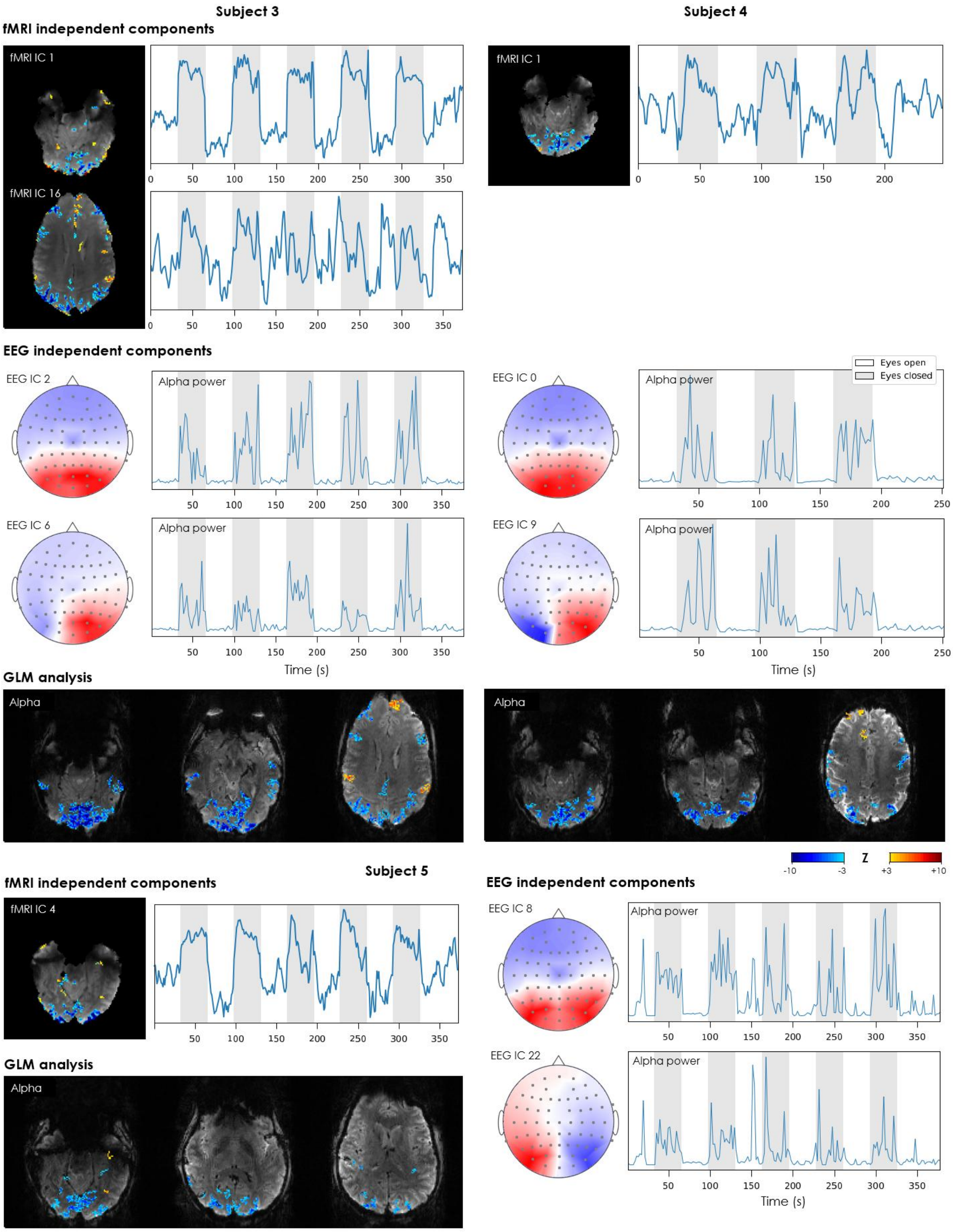

Supp. Fig. 7. Sensitivity of simultaneously-acquired EEG and fMRI signals to activity modulation from eyes closing and opening, complementing Fig. 8 with the other 3 subjects that underwent this task

(BrainCap MR7Flex sub-group). The fMRI ICs are shown in terms of their spatial distribution (converted to a Z-score map and thresholded at $|Z|>1.4$) and timecourse. To harmonize with the alpha power timecourses (EEG ICs), each map-timecourse pair of fMRI ICs is displayed with a polarity such that the timecourse correlates positively with the eyes-closed blocks (i.e. high in eyes-closed, low in eyes-open). The EEG ICs are shown in terms of their scalp distribution and the timecourse of their alpha power (7–13 Hz). The two ICs showing the strongest alpha modulations locked with the paradigm are shown. The fMRI and EEG IC timecourses and the EEG IC scalp maps are shown in arbitrary units. The GLM analysis results are shown in terms of Z-score maps for the HRF-convolved EEG-derived alpha power timecourse, thresholded at $|Z| > 3.0$, in representative slices covering the visual cortex and superior cortical areas that showed significant responses.

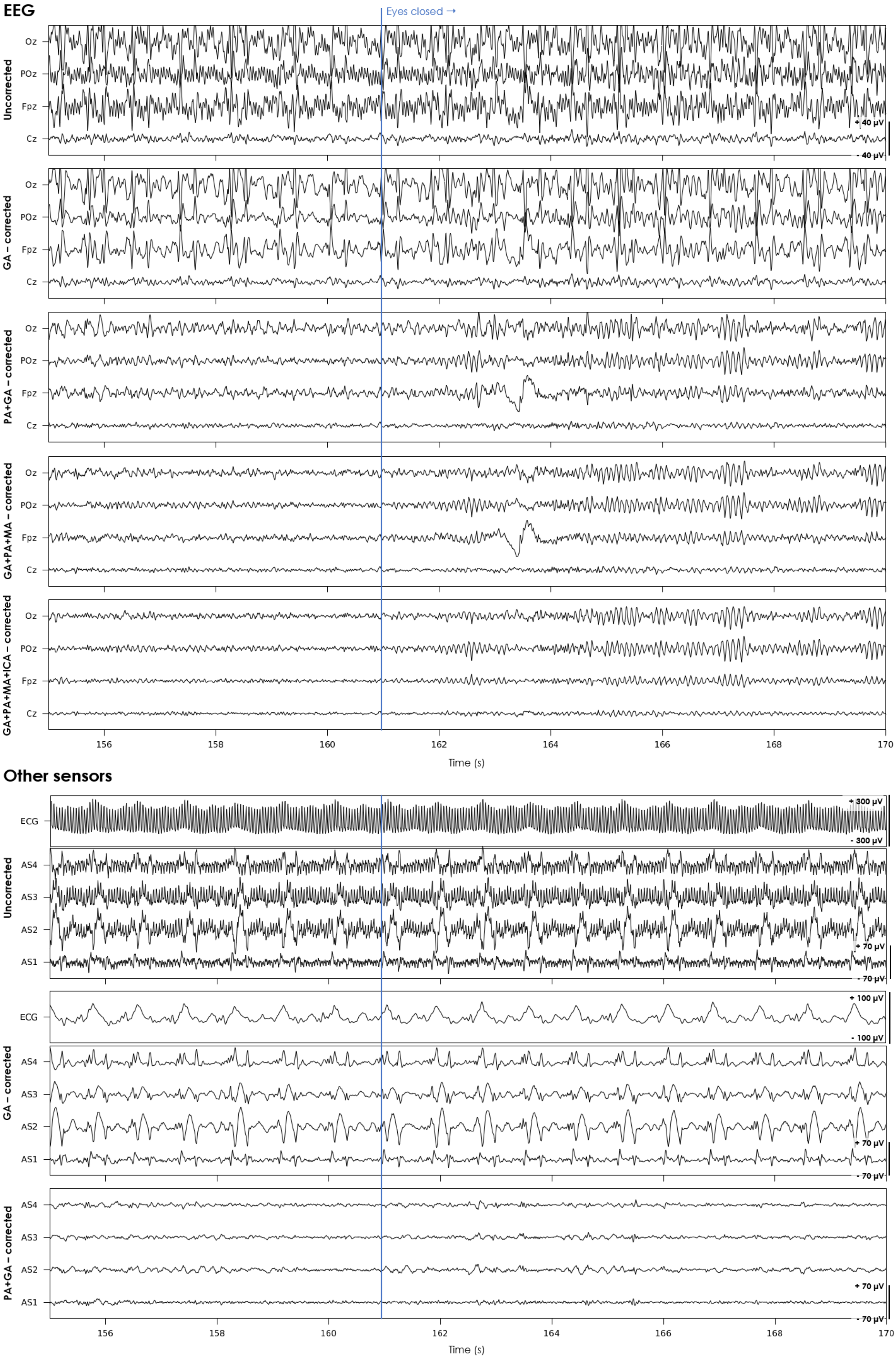
EEG
Eyes closed →
Uncorrected
GA – corrected
PA+GA – corrected
GA+PA+MA – corrected
GA+PA+MA+ICA – corrected
Oz
POz
Fpz
Cz
+ 40 µV
- 40 µV
156
158
160
162
164
166
168
170
Time (s)
Other sensors
ECG
AS4
AS3
AS2
AS1
+ 300 µV
- 300 µV
+ 70 µV
- 70 µV
+ 100 µV
- 100 µV

Supp. Fig. 8. Impact of MRI-related artifacts and correction steps on EEG traces (top) and other sensors (ECG and artifact sensors; bottom), shown for an example period from an eyes-open/closing run (concurrent 1.6 mm fMRI, BrainCap MR7Flex). The vertical blue line marks a queue to close the eyes. For consistency, all cases are shown after downsampling to 200 Hz, bandpass-filtering to 1–40 Hz, bad channel interpolation and re-referencing to the channel average; all EEG traces are plotted with the same amplitude scale (-40 to +40 µV). As can be observed, the uncorrected EEG signals are dominated by high-frequency artifacts on most channels; after GA correction, PA epochs become the dominant feature; after PA correction, the increase in alpha waves with the eyes closed now becomes readily visible in occipital channels (Oz, POz); nonetheless, the MA and ICA-based correction steps still bring improvements, reducing artifacts such as probable PA residuals and the large deflection observed in Fpz near the transition moment (likely resulting from eye movements). The fully corrected run exhibits visibly clean traces with well-preserved alpha waves, which are strongly amplified when the eyes are closed, particularly in occipital channels. We note that the ICA-based correction step performed here followed the same criteria as for the resting-state runs (described in section 2.4.1, Supp. Table II), and did not focus on selecting specifically alpha-related ICs. In addition to EEG, the other sensors are displayed up until the step where they are used for EEG correction.

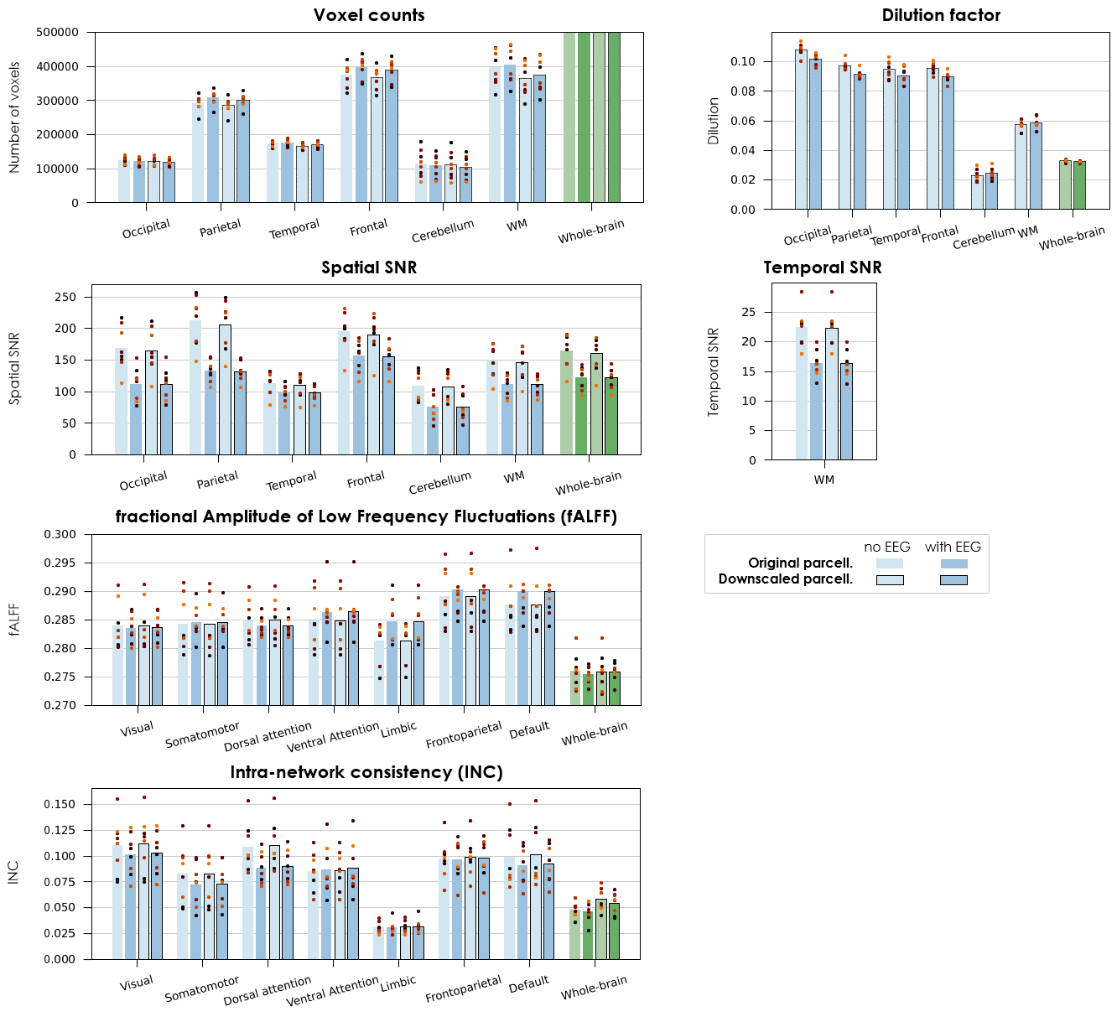

Supp. Fig. 9. Impact of partial volume effects on the evaluation of MRI data quality without and with EEG, for different brain regions and networks. This analysis was focused on the 0.8 mm GE-EPI data,

with the in-house EEG cap, and covered the main metrics for image quality and functional sensitivity considered in the study. To test the influence of partial volume effects, each of the parcellations that had been defined for the 0.8 mm data was “artificially downscaled”: the grid was split in cubes of 2×2×2 voxels, and for each cube, all voxel labels were replaced by the majority label of the cube. In this way, the parcellation became “coarser”, to a degree comparable to the 1.6 mm data and hence with similar partial volume effects, while preserving other parameters such as the voxel grid, spatial resolution and noise properties of the 0.8 mm data. The main quality metrics were then computed for these downscaled parcellations (bars with black contour), and compared with the original results (bars without contour). Two additional metrics were computed as well: (i) the number of voxels in each brain region, to assess whether certain regions tended to gain or to lose voxels in the coarser parcellation; and (ii) a “dilution factor”, computed as 1 minus the average value in each downscaled region (coarse parcellation) of an image where every voxel inside the original region (finer parcellation) is assigned the value of 1, and every voxel outside is assigned 0 – hence, the larger the amount of 0’s included, and 1’s excluded, in the coarse region, the higher the resulting dilution value. Each bar represents the average across subjects; the dot markers represent the individual subjects.

Supplementary Tables

Supp. Table I. MRI sequences and parameters used in this study

Type	Sequence	Timings (ms)	Flip angle (°)	Resolution [a] (mm)	Field of view (mm)	Readout bandwidth (Hz/Px)	Undersampling	Acq. time (min)
Structural Low SAR	3D GRE	TE/TR = 3.5/10	6	1.0	220×166×192 (axial slab)	240	2×2 GRAPPA 7/8×7/8 partial Fourier	1.3
Structural T_1-weighted	3D CS MP2RAGE (Mussard et al., 2020)	TE/TR = 2.1/6000 TI_1/TI_2 = 800/2700	4/5	0.6	240×226×154 (sagittal slab)	240	4.0× on the PE [b] plane 252 samples/TR	7.3
B_0 mapping	Double-echo 2D GRE	TE_1/TE_2/TR = 3.0/5.2/20	8	2.0	220×168×144 (72 axial slices)	630	2× GRAPPA	0.65
B_1 mapping	3D SA2RAGE (Eggenschwiler et al., 2012)	TE/TR = 1.07/2400 TD_1/TD_2 = 60/1800	4/11	2.0	256×256×144 (sagittal slab)	490	2× GRAPPA 6/8×6/8 partial Fourier	2.25
fMRI 1.6 mm	2D SMS GE-EPI (Setsompop et al., 2012)	TE/TR = 23/1050	54	1.6	208×208×134 (84 axial slices)	1924 [c]	2× GRAPPA, 4× SMS 7/8 partial Fourier	PD [b]
fMRI 0.8 mm	2D SMS GE-EPI (Setsompop et al., 2012)	TE/TR = 29/3520	81	0.8	192×192×116 (132 ax. slices)	1226 [c]	3× GRAPPA, 3× SMS 6/8 partial Fourier	PD [b]
Ref. EPI distortion correction 1.6 mm [d]	2D SE-EPI	TE/TR = 40/7310	86	1.6	208×208×134 (84 axial slices)	1924	2× GRAPPA 7/8 partial Fourier	1.5
Ref. EPI distortion correction 0.8 mm [d]	2D SE-EPI	TE/TR = 49/12170	90	0.8	192×192×116 (132 ax. slices)	1226	3× GRAPPA 6/8 partial Fourier	3.2

a. All protocols were defined with isotropic resolution; the value shown for each entry corresponds to the voxel width in all three directions.
b. PE: phase encoding; PD: paradigm-dependent (described in section 2.1.3).
c. Acquired with posterior-to-anterior PE direction.
d. SE-EPI with in-plane encoding parameters and under-sampling matched to the main fMRI protocol; comprised 3 volumes acquired in posterior-to-anterior PE direction, and 3 in the opposite sense.

Supp. Table II. EEG artifact correction approach implemented for this work

Step	Target artifact	Approach
1	(GA triggers)	From the recorded fMRI volume triggers, slice triggers were created and fine-tuned by temporal correlation as described in (Jorge et al., 2015b), after 20× temporal upsampling.
2	(PA triggers)	Initial estimates from the ECG channel (lowpass-filtered with cutoff below the slice GA fundamental frequency), manually revised, and then fine-tuned with a correlation-maximization approach using suitable EEG channels (similarly lowpass-filtered), as performed in (Jorge et al., 2019).<br>**Notes:** The quality of the ECG signal after GA filtering was, in many cases, sufficient to allow direct marking of most R-peaks. In some other cases, however, the signal was affected by stronger artifacts that frequently distorted the ECG trace, and some R-peaks could not be confidently set. The additional fine-tuning procedure using suitable EEG channels largely helped retrieving more accurate PA epochs in those instances.
3	GA	**Average artifact subtraction (AAS)** (Allen et al., 2000) applied on a volume basis, with an averaging window spanning the 100 closest epochs, followed by **optimal basis set (OBS)** removal (Niazy et al., 2005) at slice level, removing the 2 strongest components.<br>**Notes:** A preliminary analysis revealed that each slice GA waveform tended to be more similar to the same slice index in adjacent volumes than to adjacent slices in the same volume, for both fMRI protocols. Therefore, AAS was applied on a volume basis. OBS was found useful to remove remaining GA spectral content, but kept at a conservative level due to its propensity to over-correct (Jorge et al., 2019).
4	PA	**K-means clustering**-based approach validated in (Jorge et al., 2019), in line with (Gonçalves et al., 2007): for each EEG channel, the PA epochs were clustered using K-means (squared Euclidean distance), set at 12–21 clusters for each subject depending on performance. The cluster-average PA waveforms (i.e. centroids) were then subtracted from each of the epochs belonging to the respective cluster. Outlier clusters (typically with a single epoch) were assigned to the closest cluster and corrected with the respective centroid.
5	–	Temporal downsampling to 200 Hz.
6	MA	**Offline multi-channel recursive least-squares regression (oM-RLS)** using the reference artifact sensor signals from each cap variant, restricted to a spectral bandwidth of 0.75–70 Hz; a kernel of -10 to +10 temporal shifts was set for the regression model (Masterton et al., 2007).<br>**Notes:** Although we have previously employed oM-RLS restricted to a bandwidth of 0.75–35 Hz, to target MAs and PA residuals (Jorge et al., 2015a), a preliminary analysis explored in this work showed that, by expanding the bandwidth, the method was also able to reduce higher-frequency contributions such as from GA residuals and Helium coldhead artifacts.
7	–	Temporal bandpass filtering to 0.75–70 Hz (chosen as the range of interest for the quality analysis), replacement of bad channels by interpolation (0–6 per recording), and re-referencing to the channel average.
8	Various	**Temporal ICA** using the extended Infomax algorithm (Lee et al., 1999); the resulting ICs were visually inspected in terms of their topography, temporal behavior and spectral profile, to identify and exclude components associated to eye blinks, muscle activity, and residuals from gradient, pulse, motion and environment artifacts; for example, ICs dominated by spectral peaks at the GA slice frequency (20 Hz / 12.5 Hz) and harmonics were identified as residual GA artifacts. The process resulted in the exclusion of 49±4 ICs per recording across all EEG-fMRI data. In the reference group, the ICs were inspected to remove mainly eye blinks and muscle activity, resulting in the exclusion of 28±5 ICs per recording.
